# Supplementary material for: Familiarity breeds success: pairs that meet earlier experience increased breeding performance in a wild bird population
Source: Proc Biol Sci. 2020 Dec 23;287(1941):20201554. doi: 10.1098/rspb.2020.1554 (PMC7779496; doi:10.1098/rspb.2020.1554)
Supplement: Supplementary material to: Familiarity breeds success: pairs that meet earlier experience increased breeding performance in a wild bird population [file rspb20201554supp1.pdf]

Supplementary material to:

Culina, A.[1,2]*, Firth, J.A. [2,3] & Hinde, C. A.[2,4]: Familiarity breeds success: pairs that meet earlier experience increased breeding performance in a wild bird population. Proceedings B

DOI 10.1098/rspb.2020.1554

1 Netherlands Institute of Ecology, Wageningen, the Netherlands

2 Edward Grey Institute, Department of Zoology, University of Oxford, Oxford, UK.

3 Merton College, University of Oxford, Oxford, UK

4 Behavioural Ecology Research Group, Department of Biology, Anglia Ruskin University, Cambridge, United Kingdom

Consists of

1) Supplementary methods
    - I) Additional analysis on the meeting time and time of pair formation (includes figures S1 to S3)
    - II) Additional anlaysis on the robustness of the resuts
    - III) Meta – analysis on the correlation between breeding success and future mating decisions (divorce or re-mating with the same partner), includes Table S1
2) Supplementary results
    - I) Supplementary figures (S4 to S6)
    - II) Supplementary tables

1) SUPPLEMENTARY METHODS

Note that the analysis code for the supplementary analysis and results (figures) can be found at https://doi.org/10.5061/dryad.2z34tmpj9

I) Additional analysis on the meeting time and time of pair formation

Pair meting time and arrival time of male and female

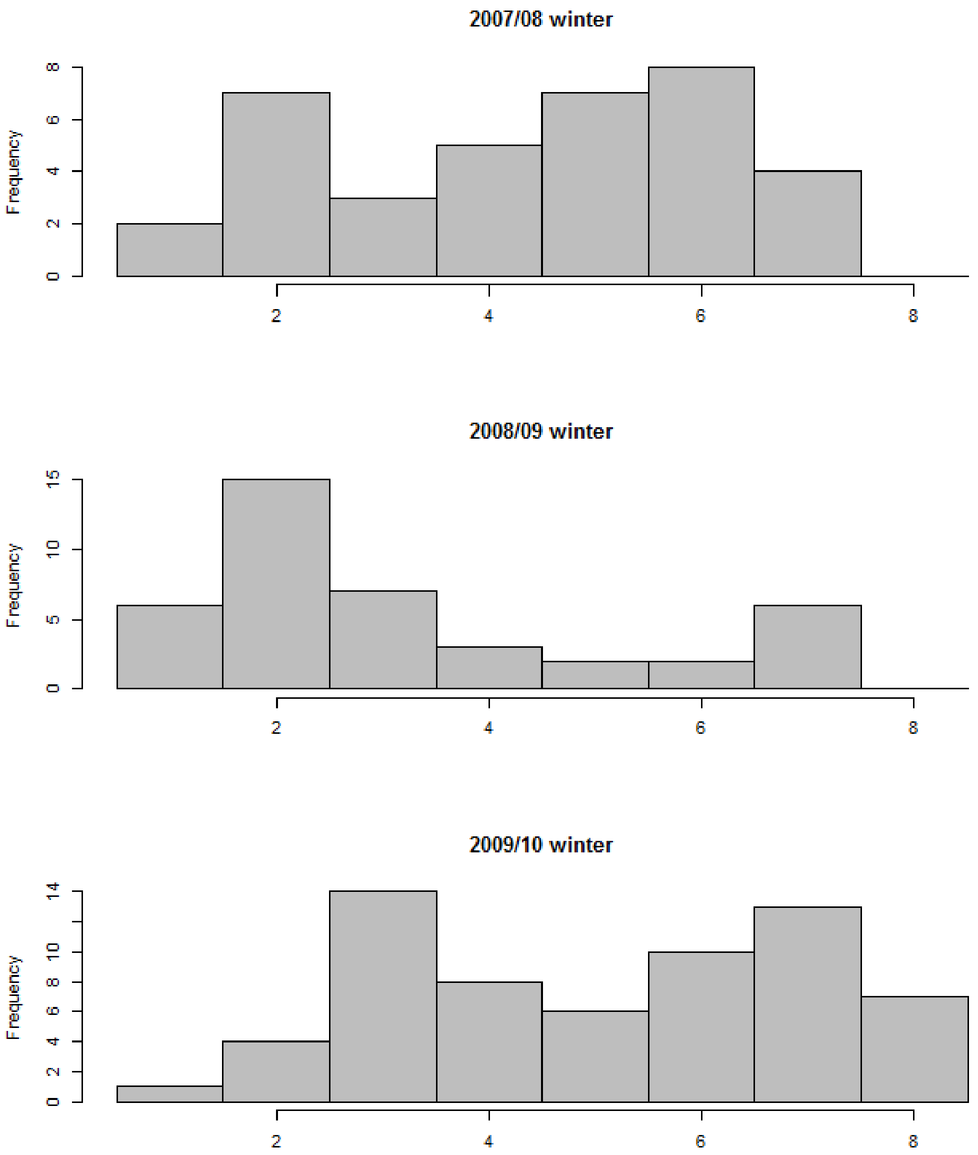

Figure S1. Histograms of the month when a female and a male of a future breeding pair (i.e. a pair that will breed for the first time in the following breeding season) were first detected in the same winter flock in the 2007 to 2009 winters. Numbers represent different months: from Aug (1) to March (8).

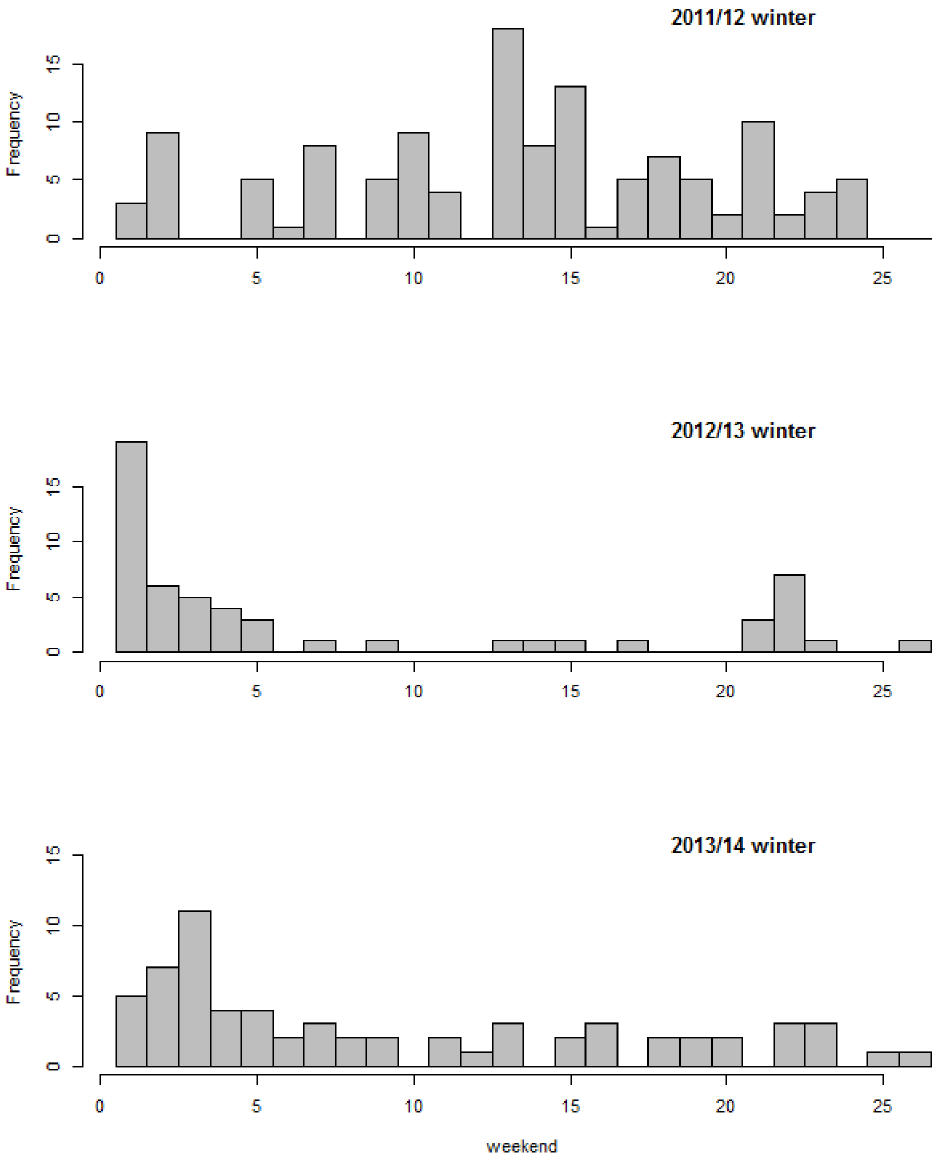

Figure S2. Histograms of the weekend when a female and a male of a future breeding pair (i.e. a pair that will breed for the first time in the following breeding season) were first detected in the same flock in winters the 2011 to 2013. Numbers represent weekend: from 2nd weekend of September to the first weekend of March.

We ran a simple linear model with the meeting time as a response variable, and female and male arrival time, their interaction, and the number of associates of a male and female as predictors. In both sets of winters (2007/08 to 2009/10 and 2011/12 to 2103/14) pairs where female and male arrived earlier have also met earlier. We repeated the analysis excluding those pairs where members have met in the same moth/weekend they have both arrived in the population, or the same month/weekend when the later arriving member arrived: 45 pairs in 2007/08 to 2009/10 winters, and 99 pairs in 2011/12 to 2013/14 winters. The effects of the arrival time of male and female stayed similar as in the previous model with all pairs.

Does meeting moth/weekend represent the start of pair bonding

Previous work on the data from the same population has shown that members of pairs that will later breed together associate more frequently and belong the same flocks more consistently than birds with a similar foraging pattern (Psorakis et al. 2012). Further, in our previous analysis on the same data we have shown that the time when a pair was first seen together could be considered as a time when a pair formation has started (Firth et al. 2018). Still we have run some additional analysis to compare the Simple Ratio Index (SRI) of a pair bond with other SRI's in the population.

First, we compared SRI of the bond with the 50th and 75th percentile of the distribution of the SRI's of all birds that were detected in the network and subsequently have been known to breed (even if they did not qualify for our analysis on meeting time and breeding success). Second, we calculated a preference for a partner in a meeting month/weekend in two ways: (1) as the difference between SRI of the pair bond and the highest SRI of the female/male in the month/weekend when a pair has met, and (2) as the difference between pair SRI and an average SRI of the female/male of a pair for that month/weekend.

In all six winters, for 2/3 of males and females a SRI with a future breeding partner was above the value of the 75th percentile of the all SRIs (for all dyads) in that month/weekend. In the first three winters, a partner was the strongest associate in the meeting month for 37 females, and 35 males (out of 140 males and females). This was true for 64 females and 71 males (out of 244) in the second set of winters.

Next, birds might be more likely to meet their partner by chance in those months/weekends when they have associated with more birds (i.e. had higher degree). However, the distribution of the degrees of males and females showed that these vary between 1 and 120 associates in a meeting month (first dataset, Fig S3) and between 1 and 77 associates in a meeting weekend (second dataset, Fig S3). Thus, it is unlikely that birds meet they partners only because they associate with a large number of individuals in a particular month/weekend. Furthermore, while the degree in a meeting month/weekend represents all the birds that a focal individual has associated with in a given month/weekend, the typical flock size the individual experiences is around five birds. Thus, it seems reasonable to assume that a bird has indeed 'met' all of the birds within the flock (including the future breeding partner).

Finally, the model on the predictors of the meeting time (the first subsection, 'Pair meting time and arrival time of male and female') did not support female or male meeting degree, nor their relative meeting degree, to influence meeting time. Note that there was only a very low effect of female meeting degree in the second set of winters (effect size = 0.007; SE = 0.0018, z= 3.861, p=0.000113).

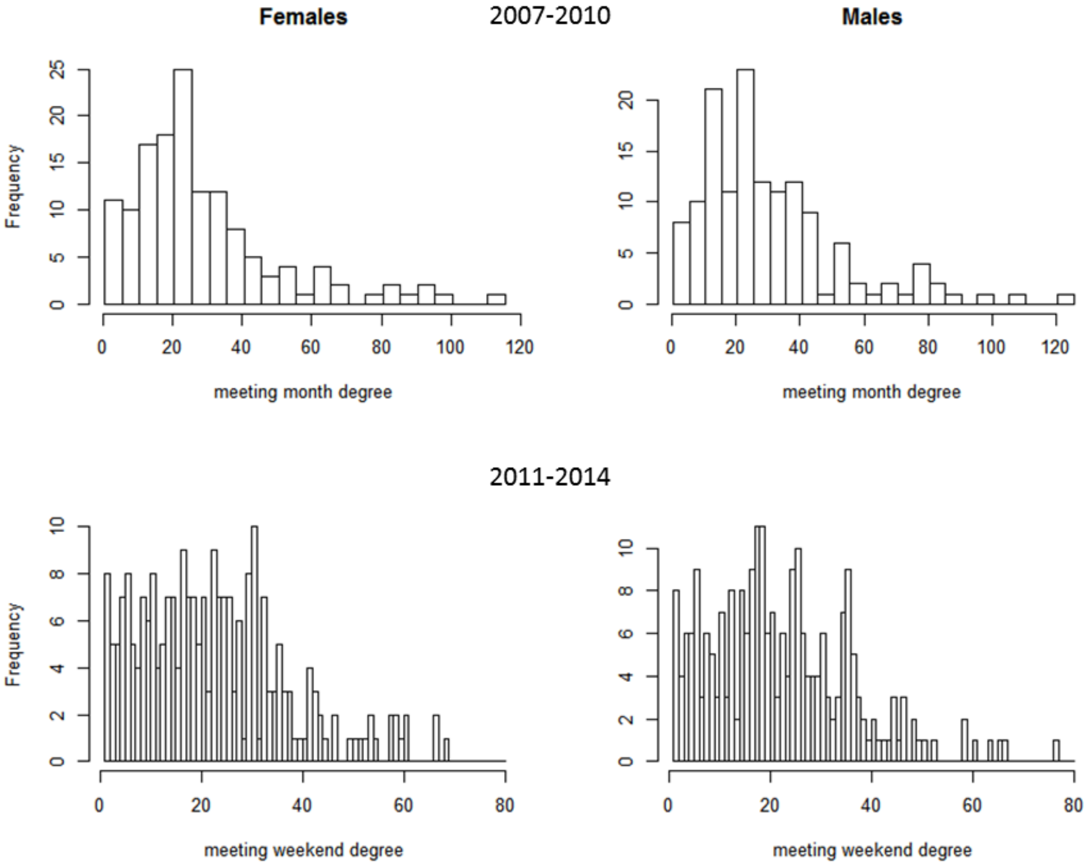

Figure S3 Degree (i.e. number of associates) of a female and a male in a month (winters 2007/08 to 2009/10) or weekend (winters 2011/12 to 2013/14) when a pair was first detected together in the same flock.

<u>Predictors of pair bond strength</u>

We have run models (separately for each set of winters) to determine what factors predict the pair bond SRI in the month/weekend when a pair has met, the overall winter SRI of a pair bond, and the relative winter SRI of the pair bond. Relative winter SRI is calculated as the winter SRI of the pair bond divided by the sum all of SRIs (with all of the winter associates) of a female/male. The relative winter bond SRIs of males and females were strongly correlated (0.788; CI: 0.716- 0.884 for 2007/08-2009/2010 winters, of 0.92; 0.98-0.93 for the 2011/12 to 2013/14 winters). In all the models we used a log transformed value of the SRI.

The model on the SRI of the meeting month/weekend included winter, relative or absolute degree of female and male in that month/weekend, and the interaction between arrival time of male, arrival time of female, and meeting time. Relative degree expresses the number of associates a bird had in the meeting month given the overall number of associates in winter. The model estimates showed that in the first set of winters, meeting SRI increased with decreasing number of male associates in that month (however, with a low effect size). In the

second set of winters none of the explanatory variables explained the variation in meeting SRI.

The models on the winter SRI and relative winter SRI of the pair included winter, female and male winter degree, and interactive effects of arrival time of male, female, and the meeting time. The results on the first three winter have showed that winter SRI was best predicted by pair meeting time: pairs that met earlier, had highest winter SRI. Further, pairs with low-degree males and high-degree females had higher SRI, however, these two effects had a very low effect size. In the second set of winters, pairs that met earlier had higher winter SRI, while the effect of male winter degree was very small. Relative winter SRI was best predicted by the female and the male winter degrees in the first three years (low effect size), and by meeting time and male winter degree in the second set of winters.

II) Additional analysis on the robustness of the results

We have run the best model obtained by the main model selection (as described in the Method section of the main text, step 1 to step 3) while controlling for several variables that could potentially influence the strength of the effect of meeting time on breeding success. Seven variables were selected to control for the possible effect of whether a pair bond has indeed started to form in the meeting month/weekend and how strong this bond was:

a) meeting month/weekend SRI of a pair bond;
b) difference between meeting month/weekend SRI and the 75th percentile of the values for all the SRI of all birds detected in the network in that month/weekend, for all the birds that have been detected to breed in the following season (no matter if the partnership was new or old).
c) difference between maximum SRI of the female/male and the bond SRI in the meeting month/weekend. This variable expresses the preference of the focal individual for her/his partner;
d) difference between average SRI of a female and the bond SRI in the meeting month/weekend. This variable also expresses the preference of the focal individual for her partner. Value for the male was not included as it was highly correlated with the value for the female (r>0.9)
e) winter SRI of the pair bond;
f) relative winter SRI of the pair bond, calculated for the female. We did not include the value for a male as it was highly correlated with the females value (r>0.79);

We further added female and male degree in the meeting month/weekend to control for the possibility that gregariousness of the individual in the moth/weekend of meeting influenced the probability that a pair has met.

III) Meta – analysis on the correlation between breeding success and future mating decisions

To decide on the component of breeding success (we term this breeding stage) that has the strongest influence on future mating decisions (divorce or stay with the same partner) we conducted a meta-analysis on the correlation between breeding success in the year *t* and the probability that a pair divorces or stays together to breed in the breeding season *t+1*. For this analysis we used a subset of data on Paridae species originally used in Culina et. al 2015 (*Parus major, Cyanistes caeruleus*, *Poecile montanus*, and *Poecile atricapillus*). The analysis included 10 papers with 63 effect sizes (Table S1). We used the 'MCMCglmm' package in R (Hadfield 2010) to conduct the analysis. We based model selection on the deviance information criterion (DIC), which is a Bayesian analogue of Akaike information criterion (AIC, Spiegelhalter et al. 2002). We considered a model that had the DIC value more than 4 units lower to be a better fit to the data than the competing model (or models). In addition, for each set of models, we also checked the significance of the fixed effects (using *P*-values provided in the MCMCglmm as pMCMC). We contrasted six different models (Table S24) in order to test whether models including stage of breeding influences the effect size. Our response variable was Fishers-Z score (normal transformation of Pearson's correlation coefficient r), and our predictor variables were: breeding stage (this can be: timing of breeding, clutch size, brood level measures, and fledgling level measures); dichotomisation of breeding success (whether or not breeding success was dichotomised); reference pairing class (i.e. whether breeding success of divorced birds was compared to faithful birds, widowed birds, or population mean). We made this choice of predictors based on Culina et al. (2015). We also included 'population' as the random effect. Population corresponded to the study (i.e. main paper) in all but one case (Dhondt et al. 1996) where two populations were included in one paper. Each analysis was run for 5,000,000 iterations, burn-in of 500,000, and thinning interval of 10. Two models gained similar (and the best support) in the set of models tested: one that included 'breeding stage' + 'reference pairing class'; and the other one 'breeding stage' + 'dichotomisation' + 'reference pairing class' (Table S20). The results have shown that laydate and clutch size were the only components of breeding success that correlated with the future divorce probability (effect size for laydate = 0.16, 95%CI = 0.07 - 0.24; for clutch size = 0.12, 95%CI = 0.04 - 0.20).

Table S1 Data on the correlation between breeding success and divorce used to perform the meta-analysis to detect which component of breeding success is a strongest predictor to divorce in four Paridae species (*Parus major*, *Cyanistes caeruleus*, *Poecile montanus*, and *Poecile atricapillus*). This data is a subset of the dataset used in the meta-analysis on divorce and breeding success published by Culina et al. 2015 (Appendix S1).

Latine name	Population	Sample size	Breeding stage	Dichotomisation	Reference pairing class	Pearson's r	Fisher's-Z	error
Poecile atricapillus	Ramsay et al., 2000	26	fledging	yes	faithful	-0.13	-0.14	0.21
Poecile atricapillus	Ramsay et al., 2000	25	clutch	no	faithful	0.10	0.10	0.21
Cyanistes caeruleus	Ramsay et al., 2000	137	timing	no	faithful	0.11	0.11	0.09
Cyanistes caeruleus	Ramsay et al., 2000	137	clutch	no	faithful	0.07	0.07	0.09
Cyanistes caeruleus	Ramsay et al., 2000	137	fledging	no	faithful	0.02	0.02	0.09
Cyanistes caeruleus	Dhondt and Adriaensen, 1994	147	clutch	no	faithful	0.23	0.24	0.08
Cyanistes caeruleus	Dhondt and Adriaensen, 1994	147	fledging	no	faithful	0.24	0.25	0.08
Cyanistes caeruleus	Dhondt and Adriaensen, 1994	147	timing	no	faithful	0.34	0.35	0.08
Cyanistes caeruleus	Garcia-Navas and Sanz, 2011	42	clutch	no	faithful	0.17	0.17	0.16
Cyanistes caeruleus	Garcia-Navas and Sanz, 2011	42	brood	no	faithful	0.11	0.11	0.16

Cyanistes caeruleus	Garcia-Navas and Sanz, 2011	42	fledging	no	faithful	0.02	0.02	0.16
Cyanistes caeruleus	Kempenaers et al., 1998	35	brood	no	faithful	-0.29	-0.30	0.18
Cyanistes caeruleus	Pampus et al., 2005	90	brood	no	faithful	-0.18	-0.18	0.11
Cyanistes caeruleus	Pampus et al., 2005	90	brood	no	faithful	-0.08	-0.08	0.11
Cyanistes caeruleus	Pampus et al., 2005	90	clutch	no	faithful	0.04	0.04	0.11
Cyanistes caeruleus	Pampus et al., 2005	90	fledging	no	faithful	0.03	0.03	0.11
Cyanistes caeruleus	Pampus et al., 2005	90	fledging	no	faithful	-0.07	-0.07	0.11
Cyanistes caeruleus	Valcu and Kempenaers, 2008	38	fledging	no	faithful	-0.01	-0.01	0.17
Cyanistes caeruleus	Valcu and Kempenaers, 2008	38	fledging	no	faithful	0.18	0.18	0.17
Cyanistes caeruleus	Valcu and Kempenaers, 2008	38	clutch	no	faithful	0.34	0.35	0.17
Cyanistes caeruleus	Valcu and Kempenaers, 2008	38	clutch	no	faithful	0.37	0.38	0.17
Cyanistes caeruleus	Valcu and Kempenaers, 2008	38	timing	no	faithful	-0.65	-0.78	0.17
Cyanistes caeruleus	Blondel et al., 2000	81	clutch	no	population mean	0.03	0.03	0.11
Cyanistes caeruleus	Blondel et al., 2000	81	timing	no	population mean	0.19	0.20	0.11

Cyanistes caeruleus	Blondel et al., 2000	81	fledging	no	population mean	-0.37	-0.39	0.11
Cyanistes caeruleus	Blondel et al., 2000	81	fledging	no	population mean	-0.35	-0.37	0.11
Cyanistes caeruleus	Dhondt and Adriaensen, 1994	61	timing	no	population mean	0.00	0.00	0.13
Parus major	Dhondt et al. 1996, population 1	57	clutch	no	faithful	0.08	0.08	0.14
Parus major	Dhondt et al. 1996, population 2	57	fledging	no	faithful	0.05	0.05	0.14
Parus major	Dhondt et al. 1996, population 2	57	timing	no	faithful	0.04	0.04	0.14
Parus major	Kempenaers et al., 1998	83	brood	yes	faithful	-0.11	-0.11	0.11
Parus major	Pampus et al., 2005	50	fledging	no	faithful	0.31	0.32	0.15
Parus major	Pampus et al., 2005	51	brood	no	faithful	-0.21	-0.21	0.14
Parus major	Pampus et al., 2005	50	clutch	no	faithful	-0.18	-0.18	0.15
Parus major	Pampus et al., 2005	50	brood	no	faithful	-0.11	-0.11	0.15
Parus major	Pampus et al., 2005	51	fledging	no	faithful	0.23	0.23	0.14
Parus major	Pampus et al., 2005	50	fledging	no	faithful	-0.03	-0.03	0.15
Parus major	Pampus et al., 2005	50	brood	no	faithful	-0.18	-0.18	0.15
Parus major	Pampus et al., 2005	51	clutch	no	faithful	0.19	0.19	0.14
Parus major	Pampus et al., 2005	51	brood	no	faithful	-0.04	-0.04	0.14
Parus major	Pampus et al., 2005	50	fledging	no	faithful	0.10	0.10	0.15
Parus major	Saitou, 2001	287	clutch	no	faithful	0.07	0.07	0.06
Parus major	Saitou, 2001	287	fledging	no	faithful	0.12	0.12	0.06
Parus major	Saitou, 2001	287	timing	no	faithful	0.00	0.00	0.06
Poecile montanus	Orell et al., 1994	223	fledging	yes	faithful	0.03	0.03	0.07

Poecile montanus	Orell et al., 1994	92	fledging	no	faithful	0.05	0.05	0.11
Poecile montanus	Orell et al., 1994	92	fledging	no	faithful	0.06	0.06	0.11
Poecile montanus	Orell et al., 1994	121	clutch	no	faithful	0.10	0.10	0.09
Poecile montanus	Orell et al., 1994	117	clutch	no	faithful	0.16	0.16	0.09
Poecile montanus	Orell et al., 1994	129	timing	no	faithful	0.22	0.22	0.09
Poecile montanus	Orell et al., 1994	127	timing	no	faithful	0.24	0.24	0.09
Poecile montanus	Orell et al., 1994	223	timing	no	faithful	0.25	0.26	0.07
Poecile montanus	Orell et al., 1994	223	timing	no	faithful	0.18	0.18	0.07
Poecile montanus	Orell et al., 1994	215	clutch	no	faithful	0.15	0.15	0.07
Poecile montanus	Orell et al., 1994	203	brood	no	faithful	0.11	0.11	0.07
Poecile montanus	Orell et al., 1994	189	fledging	no	faithful	0.04	0.04	0.07
Poecile montanus	Orell et al., 1994	66	fledging	no	widowed	-0.02	-0.02	0.13
Poecile montanus	Orell et al., 1994	89	clutch	no	widowed	0.07	0.07	0.11
Poecile montanus	Orell et al., 1994	82	clutch	no	widowed	0.13	0.14	0.11
Poecile montanus	Orell et al., 1994	64	fledging	no	widowed	-0.15	-0.15	0.13
Poecile montanus	Orell et al., 1994	93	timing	no	widowed	0.24	0.25	0.11
Poecile montanus	Orell et al., 1994	90	timing	no	widowed	0.33	0.34	0.11

176

2) SUPPLEMENTARY RESULTS

I) Supplementary Figures

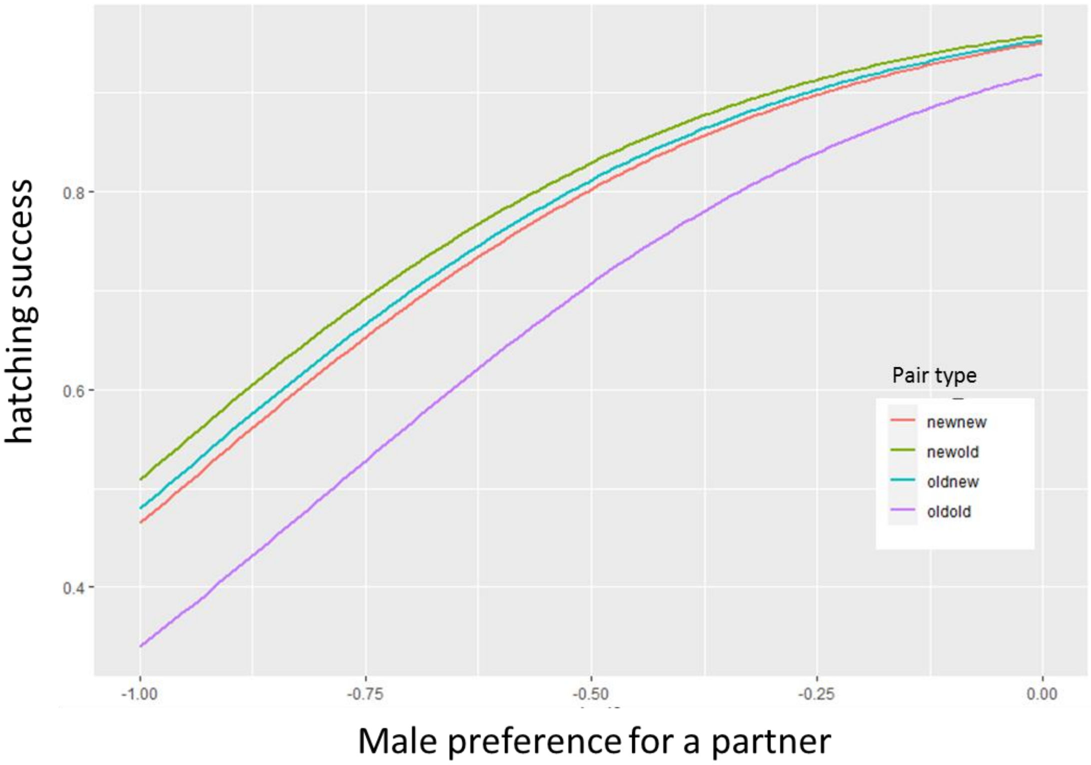

Figure S4. Hatching success as a function of male preference for the female in a month a pair has met (in the preceding winter), as estimated by the best supported model on hatching success. Preference for a partner is calculated as: (pair bond SRI – highest SRI of a male). Data come from great tit pairs breeding in Wytham woods between 2008 and 2010 breeding season. Pair type codes for whether female and male are new breeders (never bred in the populations before) or experienced (old) breeders.

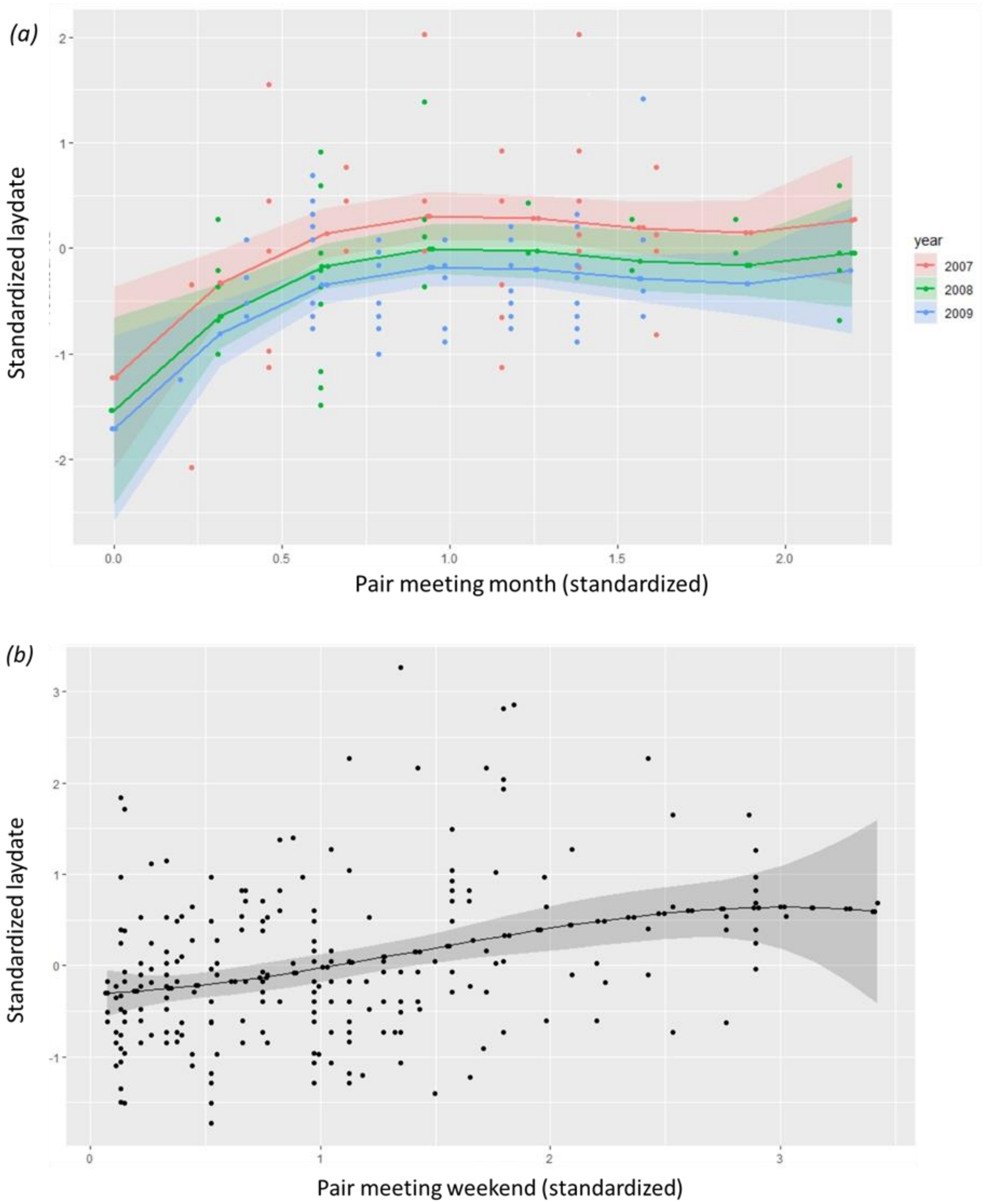

Figure S5. Standardised (for a year and for all breeding pairs) laydate of newly formed pairs of great tits as a function of standardized meeting time (for a year) of a pair in the preceding winter for a) 2008 to 2010 breeding seasons, where meeting time was a month when a pair was first seen together; b) 2012 to 2014 breeding seasons, where meeting time was a weekend when a pair was first seen together. Shaded areas represent 95% CI of the line. Dots represent the original data points.

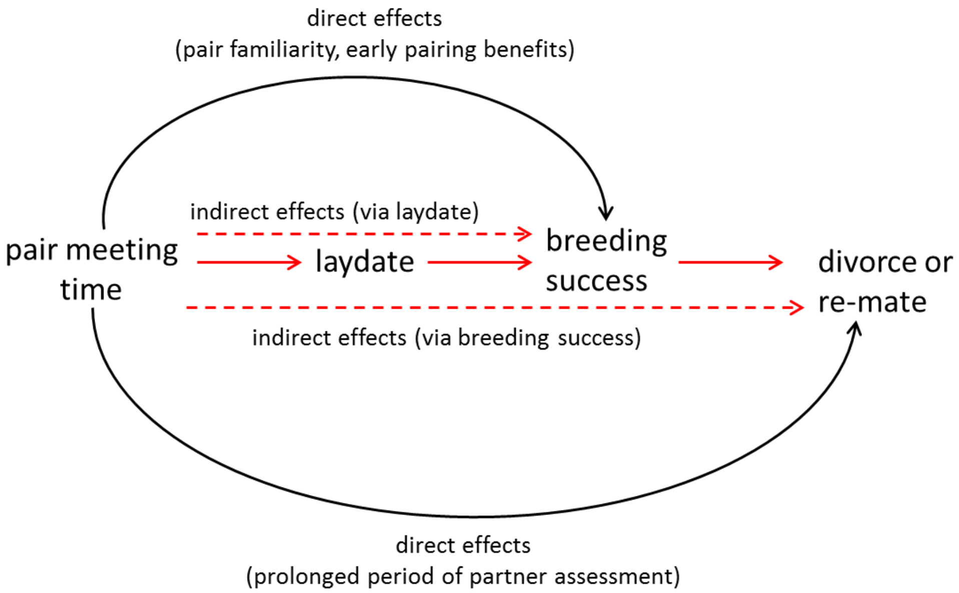

Figure S6. A schematic representation on the possible mechanisms through which meeting time can influence breeding success (i.e. components of breeding success other than laydate) and later mating decisions (i.e. breed with the same partner, or divorce a partner). If the influence is direct, this is represented by a full arrow, and if it is indirect (i.e. acting through one or more other mediators) the arrows are dashed. Red arrows present the mechanisms we have detected to act in the Wytham great tit population.

II) Supplementary Tables

Table S2. Spearman rank correlation coefficients between different components of breeding success of new pairs of great tits in breeding seasons 2008, 2009, 2010, 2012, and 2013. For fledging success (taking into account only those pairs that have fledged a chick) only correlation with laydate is provided as a Kendall’s rank tau with the p-value. Correlation coefficients are given for all pairs and for those pairs that have fledged at least one chick (in brackets). Statistically significant ($p<0.05$) correlation coefficients are marked with *.

Year	Component of breeding success	Clutch size	N of chicks	N of fledglings	Fledging success
2008	laydate	0.02 (0.06)	-0.01 (0.12)	-0.22 (-0.12)	-0.26
	clutch size	1	0.83* (0.87*)	0.35* (0.50*)	
	N of chicks	-	1	0.53* (0.60*)	
2009	laydate	-0.39* (-0.40*)	-0.61* (-0.56*)	-0.39* (-0.37*)	0.25*
	clutch size	1	0.77* (0.86*)	0.57* (0.74*)	
	N of chicks	-	1	0.81* (0.86*)	
2010	laydate	-0.36* (-0.32*)	-0.29* (-0.30*)	0.18 (0.01)	0.21*
	clutch	1	0.72* (0.69*)	0.34* (0.45*)	
	N of chicks	-	1	0.57* (0.72*)	
2012	laydate	-0.21* (-0.24)	0.05 (0.01)	0.37* (0.36*)	0.33*
	clutch size	1	0.63* (0.65*)	0.36* (0.33*)	
	N of chicks	-	1	0.54* (0.62*)	
2013	laydate	-0.05 (-0.04)	-0.03 (-0.03)	0.20* (0.15)	0.23*
	clutch size	1	0.73* (0.76*)	0.50* (0.57*)	
	N of chicks	-	1	0.56* (0.68*)	

Table S3. Model selection on the factors influencing laydate (standardised per year) of newly formed great tit pairs in Wytham woods breeding in 2008 to 2010 breeding seasons. As the best models in the first step supported the influence of meeting time, the potential quadratic and cubic effect were modelled in the second step. N = 140 pairs.

First step	Model structure	Df	AIC	AIC(meeting time standardized)
	year + meeting time × pair type	11	262,69	263,99
	meeting time + year	5	263,40	262,94
	meeting time × year	7	265,28	265,28
	year	4	266,01	NA
	year + meeting time + pair type	8	267,88	267,28
	year × meeting time + pair type	10	269,24	269,25
	year + pair type	7	269,67	NA
	meeting time	3	273,72	271,56
	meeting time + year × pair type	14	274,06	273,15
	intercept	2	274,19	NA
	meeting time × pair type	9	274,6	275,07
	year × pair type	13	276,71	NA
	meeting time + pair type	6	277,75	275,40
	pair type	5	279,58	NA
Second step				
	(meeting time)^3 + year	7	254,88	258,15
	(meeting time)^2 + year	6	256,92	259,18
	year + (meeting time)^2 × pair type	15	263,38	265,76
	year + (meeting time)^3 × pair type	19	267,29	269,99
Third step				
	(meeting time)^3 + year + f arrival time	8	255,08	NA
	(meeting time)^3 + year + m arrival time	8	256,88	NA
	(f arrival time)^3 + year	7	260,24	NA
	(f arrival time)^3 + year + meeting time	8	262,06	NA
	(m arrival time)^3 + year + meeting time	8	264,54	NA
	(f arrival time)^3 + year	7	266,49	NA

Table S4. Model selection on the factors influencing clutch size of newly formed great tit pairs in Wytham woods breeding between 2008 and 2010 breeding seasons. Laydate is controlled in all of the models (i.e. + laydate). As one of the best models in the first step supported the influence of meeting time, the potential quadratic and cubic effect were modelled in the second step. While model with the meeting time had similar AIC as the intercept model (i.e. model controlling for laydate only), the term for the influence of meeting time on clutch size was not statistically significant. N = 140 pairs.

First step	Model structure	Df	AIC
	intercept	2	603,33
	meeting time	3	604,73
	year	4	606,36
	meeting time + year	5	607,43
	pair type	5	607,98
	meeting time + pair type	6	609,36
	meeting time × year	7	610,61
	year + pair type	7	611,09
	year + meeting time + pair type	8	612,45
	meeting time × pair type	9	613,9
	year × meeting time + pair type	10	615,83
	year + meeting time × pair type	11	616,91
	year × pair type	13	620,66
	meeting time + year × pair type	14	622,32
Second step			
	(meeting time)^2	4	606,36
	(meeting time)^3	5	608,09
Third step			
	f arrival time	3	605.20
	m arrival time	3	605.33
	meeting time + m arrival time	4	606.43
	meeting time + f arrival time	4	606.66

Table S5. Model selection on the factors influencing the number of hatched young of newly formed great tit pairs in Wytham woods between 2008 and 2010 breeding seasons. Clutch size is controlled in all of the models (i.e. + clutch size). N = 140 pairs.

Model structure	Df	AIC
intercept	2	582,15
meeting time	3	584,14
year	4	585,63
meeting time + year	5	587,46
pair type	5	587,95
meeting time + pair type	6	589,95
year + pair type	7	591,21
meeting time × year	7	591,44
year + meeting time + pair type	8	593,16
meeting time × pair type	9	594,69
year × meeting time + pair type	10	597,15
year + meeting time × pair type	11	598,26
year × pair type	13	602,68
meeting time + year × pair type	14	604,61

Table S6. Model selection on the factors influencing the number of fledged young of newly formed great tit pairs in Wytham woods between 2008 and 2010 breeding seasons. Number of hatched young is controlled in all of the models (i.e. + number of hatched young). In the first set of models all pairs are included (N = 140 pairs). In the second set of models only those pairs with at least one fledged young are included (N = 118 pairs). While several models gained similar support for the full dataset, meeting time was not statistically significant in any of them.

First step	Model structure	Df	AIC
	year	4	778,43
	meeting time	3	779,78
	meeting time + year	5	779,90
	intercept	2	780,31
	year + meeting time + pair type	8	780,81
	year + pair type	7	781,23
	year + meeting time × pair type	11	781,46
	meeting time + pair type	6	782,49
	meeting time × year	7	782,63
	meeting time × pair type	9	783,36
	year × meeting time + pair type	10	783,81
	pair type	5	785,46
	year × pair type	13	789,85
	meeting time + year × pair type	14	789,95
Second step			
	meeting time ^2	4	779.32
	meeting time ^3	5	780.80
Third step			
	m arrival time	3	776.78
	meeting time + m arrival time	4	778.70
	meeting time + f arrival time	4	781.15
	f arrival time	3	781.95
Only pairs that fledged			
	intercept	2	501,89
	meeting time	3	503,32
	year	4	504,43
	meeting time + year	5	506,29
	pair type	5	507,8
	meeting time + pair type	6	509,01
	meeting time × year	7	509,52
	year + pair type	7	510,18
	year + meeting time + pair type	8	511,84
	meeting time × pair type	9	514,78
	year × meeting time + pair type	10	514,96
	year + meeting time × pair type	11	517,48

Table S7. Model selection on the factors influencing hatching success (i.e. proportion of eggs that hatched) of newly formed great tit pairs in Wytham woods between 2008 and 2010 breeding seasons. Laydate is controlled for in all of the models (i.e. + laydate). As the best models in the first step supported the influence of meeting time, the potential quadratic and cubic effect were modelled in the second step. Cubic effect were not considered when they would have introduced too many parameters for the sample size. N = 140 pairs. While the best supported models of the overall selection included meeting time, the term did not reach the statistical significance.

First step	Model structure	Df	AIC
	year + meeting time × pair type	11	375,56
	meeting time × pair type	9	376,93
	meeting time + year	5	378,08
	year + pair type	7	378,15
	year	4	378,37
	intercept	2	378,83
	year + meeting time + pair type	8	379,46
	meeting time	3	380,5
	year × pair type	13	381,44
	meeting time × year	7	381,92
	meeting time + year × pair type	14	382,52
	pair type	5	382,59
	year × meeting time + pair type	10	383,38
	meeting time + pair type	6	384,57
Second step			
	(meeting time)^2 + year	6	379.89
	(meeting time)^2 × pair type + winter	15	380.53
	(meeting time)^3+ year	7	381.76
	(meeting time)^2 × pair type	13	382.25
Third step			
	meeting time × pair type + m first seen	10	378.56
	meeting time × pair type + f first seen	10	378.87
	m first seen × pair type	9	388.33
	f first seen × pair type	9	388.68
	m first seen × pair type + meeting time	10	389.19
	f first seen × pair type + meeting time	10	390.47

Table S8. Model selection on the factors influencing fledging success (i.e. proportion of hatchlings that fledged) of newly formed great tit pairs in Wytham woods between 2008 and 2010 breeding seasons. Clutch size is controlled for in all models (i.e. + clutch size). As the best models in the first step supported the influence of meeting time, the potential quadratic and cubic effect were modelled in the second step. In the step 2 we only used winter + meeting time + pair type (as a simplified version of the best model, including all the parameters) because adding polynomials to the best supported model of the step 1 would have lead to over-parameterized structure (given the sample size). While the best supported model (step 3) included meeting time, the term has not reached the statistical significance (see table S18). N = 140 pairs.

First step	Model structure	Df	AIC
	year + meeting time × pair type	11	885,72
	meeting time + year × pair type	14	887,67
	year × pair type	13	902,75
	year + meeting time + pair type	8	904,19
	year × meeting time + pair type	10	906,67
	meeting time × pair type	9	908,75
	year + pair type	7	922,5
	meeting time + year	5	923,12
	meeting time + pair type	6	924,43
	meeting time × year	7	924,54
	year	4	929,92
	meeting time	3	937,06
	Intercept	2	959,54
	pair type	5	962,31
Second step			
	year + (meeting time) ^2 + pair type	9	893.81
	year + (meeting time) ^3 + pair type	10	895.81
Third step			
	year + m arrival time × pair type + meeting time	12	882.12
	year + meeting time × pair type + f arrival time	12	883.19
	year + m arrival time × pair type	11	885.77
	year + meeting time × pair type + m arrival time	12	886.03
	year + f arrival time × pair type + meeting time	12	892.95
	year + f arrival time × pair type	11	920.85

Table S9. Model selection on the factors influencing fledging success (i.e. proportion of hatchlings that fledged) of newly formed great tit pairs in Wytham woods that fledged at least one young between 2008 and 2010 breeding seasons. Clutch size is controlled for in all models (i.e. + clutch size) As the best models in the first step supported the influence of meeting time, the potential quadratic and cubic effect were modelled in the second step, however, using a simpler version of the model without interactions (otherwise the number of parameters to estimate would be too high given the sample size). N = 118 pairs.

First step	Model structure	Df	AIC
	year × meeting time + pair type	10	295,69
	year + meeting time × pair type	11	296,55
	meeting time × pair type	9	302,1
	meeting time × year	7	302,55
	year + meeting time + pair type	8	304,06
	meeting time + pair type	6	307,36
	meeting time + year	5	307,44
	meeting time	3	308,25
	year	4	308,68
	year + pair type	7	309,02
	intercept	2	316,7
	pair type	5	320,3
Second step			
	(meeting time)^2 + year + pair type	9	304,75
	(meeting time)^3 + year + pair type	10	306,22

Table S10. Model selection on the factors influencing binary fledging success (i.e. fledged at least one young vs. no young fledged) of newly formed great tit pairs in Wytham woods between 2008 and 2010 breeding seasons. Laydate is controlled in all of the models (i.e. + laydate). As one of the best models in the first step supported the influence of meeting time, the potential quadratic and cubic effect were modelled in the second step. While the best models included those with meeting time, the term did not reach statistical significance. N = 140 pairs.

First step	Model structure	Df	AIC
	meeting time	3	126,77
	year	4	127,90
	meeting time + year	5	129,61
	meeting time + pair type	6	130,04
	pair type	5	130,35
	year + pair type	7	131,53
	year + meeting time + pair type	8	132,29
	meeting time × year	7	132,66
	meeting time × pair type	9	132,7
	year + meeting time × pair type	11	134,98
	intercept	10	135,6
	year × meeting time + pair type	10	135,6
	year × pair type	13	136,53
	meeting time + year × pair type	14	137,83
Second step			
	(meeting time)^2	4	127.26
	(meeting time)^3	5	129.22
Third step			
	m arrival time	3	125.81
	f arrival time	3	127.37
	meeting time + m arrival time	4	127.79
	meeting time + f arrival time	4	128.70

Table S11. Model selection on the factors influencing laydate (standardised per year) of newly formed great tit pairs in Wytham woods in 2012 to 2014 breeding seasons. As the best models in the first step supported the influence of meeting time, the potential quadratic and cubic effect were modelled in the second step. N = 243 pairs.

First step	Model structure	Df	AIC	AIC (standardized meeting time)
	meeting time + year	5	586,20	588,71
	meeting time	3	586,89	585,77
	meeting time × year	7	590,04	590,04
	year + meeting time + pair type	8	590,15	592,42
	meeting time + pair type	6	591,32	589,62
	year × meeting time + pair type	10	593,98	593,98
	year + meeting time × pair type	11	595,15	598,15
	meeting time × pair type	9	596,33	595,34
	meeting time + year × pair type	14	598,00	611,43
	meeting time × year + meeting × pair type	13	598,88	616,91
	meeting time × year + pair type × winter	16	601,57	601,57
	pair type × year + meeting time × pair type	17	602,86	605,34
	pair type	5	608,63	NA
	intercept	2	610,26	NA
	year + pair type	7	611,43	NA
	year	4	613,31	NA
	year × pair type	13	616,91	NA
Second step				
	(meeting time)^3	5	579.92	589,37
	(meeting time)^3 + year	7	581.58	584,55
	(meeting time)^2	4	582.87	587,76
	(meeting time)^2 + year	6	585.24	590,57
Third step				
	(meeting time)^3 + m arrival time	6	581.74	
	(meeting time)^3 + f arrival time	6	581.75	
	(m arrival time)^3 + meeting time	6	587.56	
	(f arrival time)^3 + meeting time	6	592.57	
	(m arrival time)^3	5	599.76	
	(f arrival time)^3	5	604.06	

Table S12. Model selection on the factors influencing clutch size of newly formed great tit pairs in Wytham woods in 2012 to 2014 breeding seasons. Laydate is controlled in all of the models (i.e. + laydate). Term meeting time does not reach significance in the meeting time+winter, nor meeting time model. N = 243 pairs.

First step	Model structure	Df	AIC
	year	4	1017
	intercept	2	1017,66
	meeting time + year	5	1018,45
	meeting time	3	1019,26
	meeting time × year	7	1021,49
	year + pair type	7	1022,38
	pair type	5	1022,47
	year + meeting time + pair type	8	1024,06
	meeting time + pair type	6	1024,33
	year × meeting time + pair type	10	1026,82
	year + meeting time × pair type	11	1029,68
	meeting time × pair type	9	1030,03
	meeting time × year + meeting × pair type	13	1032,52
	year × pair type	13	1033,08
	meeting time + year × pair type	14	1034,68
	meeting time × winter + pair type × year	16	1037,33
Second step			
	(meeting time^2) + year	6	1020.30
	(meeting time^3) + year	7	1021.64
Third step			
	m arrival time + year	5	1018.79
	f arrival time + year	5	1018.93
	f arrival time + year + meeting time	4	1021.04
	m arrival time + year + meeting time	4	1021.20

Table S13. Model selection on the factors influencing the number of hatched young of newly formed great tit pairs in Wytham woods in 2012 to 2014 breeding seasons. Clutch size is controlled for in all of the models (i.e. + clutch size). N = 243 pairs.

Model structure	Df	AIC
year	4	1000,77
intercept	2	1001,83
meeting time + year	5	1002,12
meeting time	3	1003,82
year + pair type	7	1005,39
pair type	5	1005,81
meeting time × year	7	1005,91
year + meeting time + pair type	8	1006,59
meeting time + pair type	6	1007,69
year × meeting time + pair type	10	1010,28
year + meeting time × pair type	11	1012,28
meeting time × pair type	9	1013,59
meeting time × year + meeting × pair type	13	1016,06
year × pair type	13	1016,46
meeting time + year × pair type	14	1017,94
meeting time × year + pair type × year	16	1021,66
pair type × winter + meeting time × pair type	17	1023,73

Table S14. Model selection on the factors influencing the number of fledged young of newly formed great tit pairs in Wytham woods in 2012 to 2014 breeding seasons. Number of hatched young is controlled for in all of the models (i.e. + number of hatched young). Because the best model in the first step included meeting time, we proceeded to model polynomial effects of meeting time. Although meeting time is amongst the models with the lowest AIC, the term does not reach statistical significance. N = 243 pairs for all pairs. N = 210 for those that fledged at least one young.

All pairs			
First step	Model structure	Df	AIC
	meeting time + year	5	1150,37
	year	4	1150,58
	intercept	2	1151,11
	meeting time	3	1152,3
	meeting time × year	7	1152,41
	year + meeting time + pair type	8	1152,49
	meeting time + pair type	6	1154,04
	pair type	5	1154,27
	year + pair type	7	1154,35
	year × meeting time + pair type	10	1154,69
	year + meeting time × pair type	11	1157,05
	meeting time × pair type	9	1158,32

	meeting time × year + meeting × pair type	13	1159,55
	meeting time + year × pair type	14	1162,14
	year × pair type	13	1163,55
	meeting time × year + pair type × year	16	1164,55
	pair type × year + meeting time × pair type	17	1166,81
Second step			
	(meeting time)^2 + year	6	1152.22
	(meeting time)^3 + year	7	1154.17
Third step			
	m arrival time + year	5	1149.40
	m arrival time + year + meeting time	6	1151.03
	f arrival time + year	5	1151.97
	f arrival time + year + meeting time	6	1152.35
Pairs that fledged > 0			
	intercept	2	829.77
	meeting time	3	831.76
	year	4	832.88
	meeting time + year	5	834.87
	pair type	5	834.95
	meeting time + pair type	6	836.73
	year + pair type	7	837.92
	meeting time × winter	7	838.43
	year + meeting time + pair type	8	839.72
	meeting time × pair type	9	842.65
	year × meeting time + pair type	10	843.32
	year + meeting time × pair type	11	845.55
	year × pair type	13	848.87
	meeting time × year + meeting × pair type	13	849.18
	meeting time + year × pair type	14	850.69

Table S15. Model selection on the factors influencing hatching success (i.e. proportion of eggs that hatched) of newly formed great tit pairs in Wytham woods in 2012 to 2014 breeding seasons. Standardized laydate and clutch size is controlled for in all the models (i.e. +laydate + clutch size). Because the best model in the first step included meeting time, we proceeded to model polynomial effects of meeting time. N = 243 pairs for all pairs.

First step	Model structure	Df	AIC
	year × meeting time + pair type	10	809,11
	year + meeting time + pair type	8	809,20
	meeting time + year	5	811,46
	year + meeting time × pair type	11	811,48
	meeting time × year	7	812,32
	meeting time × year + meeting × pair type	13	812,63
	year + pair type	7	813,07
	year	4	814,93
	meeting time × year + pair type × winter	16	816,26
	meeting time + year × pair type	14	816,34
	year × pair type	13	818,91
	pair type × year + meeting time × pair type	17	819,86
	pair type	5	835,86
	meeting time + pair type	6	837,06
	intercept	2	841,1
	meeting time × pair type	9	841,93
	meeting time	3	843,01
Second step			
	year × (meeting time)^2 + pair type	13	799.19
	year × (meeting time)^3 + pair type	16	799.52
	year + (meeting time)^2 + pair type	9	810.47
	year + (meeting time)^3 + pair type	10	812.42
Thirds step			
	year × (m arrival time)^2 + pair type	13	788.70
	year × (m arrival time)^2 + pair type + meeting time	14	790.22
	year × (meeting time)^2 + pair type + m arrival time	14	796.33
	year × (f arrival time)^2 + pair type	13	796.11
	year × (f arrival time)^2 + pair type + meeting time	14	797.35
	year × (meeting time)^2 + pair type + f arrival time	14	798.85

Table S16. Model selection on the factors influencing fledging success (i.e. proportion of hatchlings that fledged) of newly formed great tit pairs in Wytham woods in 2012 to 2014 breeding seasons. Standardized laydate and clutch size are controlled for in all of the models (i.e. + laydate + clutch size). Because the best model in the first step included meeting time, we proceeded to model polynomial effects of meeting time. N = 243 pairs for all pairs. N = 210 for those that fledged at least one young.

All pairs			
First step	Model structure	Df	AIC
	year × meeting time + pair type	11	1194.72
	meeting time × year + meeting × pair type	14	1196.31
	meeting time × year + pair type × winter	17	1197.35
	year + meeting time + pair type	9	1198.46
	meeting time + year × pair type	15	1199.07
	pair type × year + meeting time × pair type	18	1199.21
	year + meeting time × pair type	12	1200.20
	year + pair type	8	1201.46
	year × pair type	14	1201.63
	meeting time × year	8	1210.01
	pair type	6	1212.94
	meeting time + pair type	7	1212.99
	year	5	1213.63
	meeting time × pair type	10	1213.99
	meeting time + year	6	1214.34
	intercept	3	1228.51
	meeting time	4	1230.51
Second step			
	year × (meeting time)^2 + pair type	14	1185.21
	year × (meeting time)^3 + pair type	17	1189.96
Third step			
	year × (f arrival time)^2 + pair type + meeting time	15	1180.29
	year × (f arrival time)^2 + pair type	14	1184.88
	year × (meeting time)^2 + pair type + f arrival time	15	1185.40
	year × (meeting time)^2 + pair type + m arrival time	15	1187.04
	winter × (m arrival time)^2 + pair type	14	1205.26
	year × (m arrival time)^2 + pair type + meeting time	15	1206.31
Pairs that fledged > 0			
	year + pair type	8	600.71
	year × pair type	14	601.83
	year + meeting time + pair type	9	602.37
	meeting time + year × pair type	15	603.45
	pair type	6	603.86
	year × meeting time + pair type	11	604.85

	year + meeting time × pair type	12	605.36
	meeting time + pair type	7	605.80
	meeting time × year + meeting × pair type	14	607.75
	meeting time × pair type	10	610.02
	meeting time + year	6	613.62
	year	5	615.08
	meeting time	4	615.40
	intercept	3	616.07
	meeting time × year	8	616.11

Table S17. Model selection on the factors influencing binary fledging success (i.e. fledged at least one young vs. no young fledged) of newly formed great tit pairs in Wytham woods in 2012 to 2014 breeding seasons. Laydate is controlled in all of the models (i.e. + laydate). Although meeting time is in the structure of one of the three best supported models, the term does not reach statistical significance. N = 243 pairs for all pairs. N = 210 for those that fledged at least one young.

All pairs			
First step	Model structure	Df	AIC
	year	4	192,25
	meeting time + year	5	193,29
	intercept	2	194,83
	meeting time × year	7	196,56
	meeting time	3	196,83
	year + pair type	7	196,99
	year + meeting time + pair type	8	197,6
	pair type	5	198,78
	year + meeting time × pair type	11	200,05
	meeting time + pair type	6	200,66
	year × meeting time + pair type	10	200,89
	meeting time × year + meeting × pair type	13	203,41
	meeting time × pair type	9	204,33
	year × pair type	13	205,79
	meeting time + year × pair type	14	206,76
	pair type × year + meeting time × pair type	17	209,39
	meeting time × year + pair type × year	16	210,43
Second step			
	(meeting time)^3 + year	6	192.85
	(meeting time)^2 + year	7	194.62
Third step			
	m arrival time + year	5	192.61
	f arrival time + year	5	194.00
	m arrival time + year + meeting time	6	194.00
	f arrival time + year + meeting time	6	195.27

Table S18. The estimates, standard errors, and test statistics of the best supported models from the main model selection, and model selection on the standardized laydate, where meeting time is also standardized (per year), for 2007-2010 dataset. When more models had similar AIC, the model with the lowest number of parameters was chosen as the best supported.

Response var.	Parameter	Estimate	Std. Error	t value/ z value	Pr(>|t|) Pr(>|z|)	disspersion parameter
**Clutch size**	(Intercept)	2.178	0.015	149.037	<2e-16	0.247
	laydate	-0.067	0.022	-2.995	0.0032	
**N hatched**	(Intercept)	1.049	0.077	13.50	<2e-16	0.1832
	clutchsz	0.116	0.008	13.88	<2e-16	
**N fledged**	(Intercept)	0.902	0.204	4.427	<e-05	1.375
	n_chicks	0.132	0.022	6.087	<e-08	
	m arrival time	-0.048	0.024	-1.972	0.0506	
**Hatch succ**	(Intercept)	2.092	1.088	1.923	0.0566	1.827
	meeting time	0.060	0.184	0.329	0.7425	
	newold pair	-1.252	1.327	-0.943	0.3475	
	oldnew pair	1.569	1.355	1.158	0.2489	
	oldold pair	0.026	1.189	0.022	0.9825	
	laydate	-0.430	0.209	-2.054	0.0420	
	meeting time:newold	0.288	0.265	1.085	0.2801	
	meeting time:oldnew	-0.319	0.237	-1.347	0.1803	
	meeting time:oldold	-0.053	0.221	-0.241	0.8098	
**Fledg succ**	(Intercept)	4.936	1.941	2.542	0.0122	6.733
	m arrvial	0.067	0.216	0.310	0.7574	
	newold pair	-0.385	1.478	-0.261	0.7948	
	oldnew pair	0.465	1.493	0.312	0.7556	
	oldold pair	0.668	1.347	0.496	0.6210	
	year2008	0.444	0.680	0.652	0.5153	
	year2009	-0.397	0.585	-0.678	0.4987	
	meeting time	-0.130	0.141	-0.920	0.3592	
	clutchsz	-0.282	0.148	-1.909	0.0585	
	m arrival:newold	0.071	0.463	0.153	0.8785	
	m arrival:oldnew	-0.251	0.324	-0.776	0.4392	
	m arrival:oldold	-0.668	0.391	-1.707	0.0902	
**Std. laydate**	(Intercept)	-12.241	0.4392	-2.787	0.0060	
	standardized meeting time	36.422	13.981	2.605	0.0102	
	I(standardized meeting time ^2)	-27.369	12.948	-2.114	0.036	
	I(standardized meeting time ^3)	0.6318	0.3586	1.762	0.080	
	year2008	-0.3125	0.1459	-2.142	0.0339	
	year2009	-0.4820	0.1242	-3.881	0.0001	

Table S19. The estimates, standard errors, and test statistics of the best supported models from the sensitivity analysis for 2007-2010 dataset. When more models had similar AIC, the model with the lowest number of parameters was chosen as the best supported.

Response var.	Parameter	Estimate	Std. Error	z value	Pr(>|z|)	disspersion parameter
**Hatch succ**	(Intercept)	3.062	1.105	2.772	0.00639	1.736
	laydate	-0.339	0.207	-1.637	0.10415	
	newold pair	-1.153	1.336	-0.863	0.38946	
	oldnew pair	1.171	1.326	0.883	0.37894	
	oldold pair	-0.706	1.185	-0.596	0.55232	
	meeting time	-0.039	0.182	-0.218	0.82799	
	m partner preference	3.082	0.925	3.332	0.00112	
	meeting time:newold	0.305	0.274	1.118	0.26576	
	meeting time:oldnew	-0.255	0.232	-1.103	0.27222	
	meeting time:oldold	0.042	0.218	0.196	0.84459	
**Fledg succ**	(Intercept)	4.847	2.006	2.415	0.0171	7.122
	m degree	0.011	0.011	1.005	0.3167	
	meeting time	-0.170	0.149	-1.141	0.2559	
	m arrival	0.058	0.226	0.262	0.7937	
	newold pair	-0.402	1.515	-0.265	0.7911	
	oldnew pair	0.643	1.559	0.412	0.6809	
	oldold pair	0.922	1.407	0.655	0.5135	
	year2008	0.421	0.699	0.602	0.5485	
	year2009	-0.251	0.621	-0.405	0.6861	
	clutchsz	-0.297	0.153	-1.934	0.0553	
	m arrival:newold	0.115	0.473	0.243	0.8083	
	m arrival:oldnew	-0.293	0.338	-0.866	0.3879	
	m arrival:oldold	-0.760	0.413	-1.838	0.0684	

Table S20. The estimates, standard errors, and test statistics of the best supported models from the main model selection, and model selection on the standardized laydate, where meeting time is also standardized (per year), for 2011-2014 dataset. When more models had similar AIC, the model with the lowest number of parameters was chosen as the best supported.

Response var.	Parameter	Estimate	Std. Error	t value	Pr(>|t|)	Disspersion parameter
**Clutch size**	(Intercept)	2.044	0.012	166.707	< 2e-16	0.282
	laydate	-0.053	0.015	-3.553	0.0004	
**N hatched**	(Intercept)	0.945	0.081	11.66	<2e-16	0.354
	clutchsz	0.118	0.010	11.87	<2e-16	
**N fledged**	(Intercept)	0.569	0.119	4.754	<2e-06	1.069
	n_chicks	0.148	0.016	8.973	< 2e-16	
**Hatch suscc**	(Intercept)	1.147	0.276	4.159	<2e-05	1.912
	m arrival time	0.051	0.062	0.830	0.4073	
	year2012	1.142	0.613	1.860	0.0641	
	year2013	-0.524	0.522	-1.005	0.3160	
	newold pair	-0.157	0.294	-0.535	0.5931	
	oldnew pair	-0.335	0.234	-1.433	0.1531	
	oldold pair	0.173	0.268	0.648	0.5179	
	laydate	0.110	0.116	0.943	0.3468	
	m arrival time: year2012	-0.192	0.482	-0.399	0.6903	
	m arrival time: year2013	0.538	0.184	2.913	0.0039	
	year2011:I(m arrival time^2)	-0.001	0.003	-0.355	0.7229	
	year2012:I(m arrival time^2)	0.010	0.052	0.198	0.8428	
	year2013:I(m arrival time^2)	-0.022	0.006	-3.436	0.0007	
**Fledg succ**	(Intercept)	-1.074	0.930	-1.155	0.2493	4.756
	laydate	0.742	0.202	3.669	0.0003	
	clutchsz	0.175	0.096	1.814	0.0709	
	year2012	0.5294	0.756	0.700	0.4845	
	year2013	1.2544	0.762	1.645	0.1012	
	f arrival time	0.0739	0.103	0.711	0.4778	
	newold pair	-0.188	0.423	-0.445	0.6566	
	oldnew pair	0.175	0.380	0.462	0.6448	
	oldold pair	0.582	0.378	1.537	0.1256	
	meeting time	0.031	0.027	1.152	0.2507	
	year2012:f arrival time	0.250	0.349	0.718	0.4734	
	year2013:f arrival time	-0.446	0.216	-2.064	0.0401	
	year2011:I(f arrival time^2)	-0.003	0.004	-0.850	0.3963	
	year2012:I(f_arrival time^2)	-0.015	0.015	-1.050	0.2950	
	year2013:I(f arrival time^2)	0.018	0.010	1.677	0.0950	
**Std. laydate**	(Intercept)	-0.366	0.085	-4.290	2.59e-05	
	standardized meeting time	0.358	0.068	5.269	3.04e-07	

Table S21. The estimates, standard errors, and test statistics of the best supported models after the sensitivity analysis for 2011-2014 dataset. When more models had similar AIC, the model with the lowest number of parameters was chosen as the best supported.

Response var	Parameter	Estimate	Std. Error	t value	Pr(>|t|)
**laydate**	(Intercept)	-0.152	0.191	-0.794	0.42781
	meeting time	0.116	0.069	1.672	0.09582
	winter SRI partner	-1.385	0.429	-3.222	0.00145
	I(meeting timer^2)	-0.011	0.006	-1.684	0.09356
	I(meeting time^3)	0.0003	0.0001	2.015	0.04498

Table S22

Table S22. Model selection on the divorce probability of a pair between the breeding seasons *t* and *t*+1, in relation to their meeting time in the winter preceding the breeding season *t*, and clutch size or the laydate of a pair in *t* for 2007/08 to 2009/10 dataset. All the models are glm's with a binomial error structure.

Model structure, with binomial errors	DIC	Df
laydate	16.67	2
meeting time + laydate	18.31	3
meeting time	22.71	2
meeting time + clutch size	23.60	3
clutch size	22.85	2
intercept only	24.07	1

Table S23. Model selection on the divorce probability of a pair between the breeding seasons *t* and *t*+1, in relation to their meeting time in the winter preceding the breeding season *t*, and clutch size or laydate of a pair in t for the 2011/12 to 2013/14 dataset. All the models are glm's with a binomial error structure.

Model structure, with binomial errors	DIC	Df
intercept only	57.12	1
laydate	57.65	2
meeting time	57.78	2
clutch size	59.04	2
meeting time + clutch size	59.78	3
meeting time + laydate	59.09	3
year	60.76	3
year + laydate	61.03	4
meeting time + year	61.69	4
clutch size + year	62.66	4
clutch size + meeting time + year	63.69	5
laydate + meeting time + year	62.80	5

Table S24. Model summary of MCMC modelling of the effect size of the relationship between breeding success before divorce and divorce. All models have the same random-effect structure ('population'). Breeding stage represents the stage of breeding cycle.

Fixed-effect structure of the model	DIC	Delta DIC
breeding stage + reference pairing class	-120.27	0
breeding stage + dichotomisation + reference pairing class	-117.56	2.27
breeding stage	-98.45	21.82
dichotomisation + reference pairing class	-95.32	24.95
reference pairing class	-91.84	28.43
intercept only	-75.03	45.24

References

Blondel J, Perret P, Galan MJ 2000 High divorce rates in Corsican blue tits: how to choose a better option in a harsh environment. *Oikos* **89**, 451–460.

Culina A, Radersma R., Sheldon BC. 2015 Trading up – fitness consequences of divorce in monogamous birds. *Biol. Reviw.* **90**, 1015-1034.

Dhondt AA, Adriaensen, F. 1994 Causes and effects of divorce in the Blue tit *Parus caeruleus*. *J. Anim. Ecol.* **63**, 979–987.

Dhondt AA, Adriaensen F, Plompen W. 1996. Beetween and within-population variation in mate fidelity in the Great Tit. Pages 235-248 in J. M. Black, editor. Partnershipsin birds.The study of monogamy. Oxford Univ. Press.

Firth JA, Cole EF, Ioannou CC, Quinn JL, Aplin LM, Culina A, McMahon K, Sheldon BC. 2018 Personality shapes pair bonding in a wild bird social system. Nat. Ecol. Evol. 2, 1696-1699.

Garcia-Navas V, Sanz, JJ. 2011 Females call the shots: breeding dispersal and divorce in Blue tits. *Behav.l Ecol.* **22**, 932–939.

Hadfield JD. 2010. MCMC Methods for Multi-Response Generalized Linear Mixed Models: The MCMCglmm R Package. *Journal of Statistical Software* **33**, 1-22.

Kempenaers B, Adriaensen F, Dhondt, AA. 1998 Inbreeding and divorce in blue and great tits. *Anim. Behav.* **56**, 737–740.

Orell M, Rytkonen S, Koivula, K. 1994 Causes of divorce in the monogamous Willow tit, *Parus montanus*, and consequences for reproductive success. *Anim. Behav.* **48**, 1143–1154.

Pampus M, Schmidt KH, Wiltschko W. 2005 Pair bond and breeding success in Blue tits *Parus caeruleus* and Great tits *Parus major*. *Ibis* **147**, 92–108.

Psorakis I, Roberts SJ, Rezek I, Sheldon BC. 2012 Inferring social network structure in ecological systems from spatio-temporal data streams. *J. R. Soc. Interface* **76**, 3055-3066.

Ramsay SM, Otter KA, Mennill DJ, Ratcliffe LM, Boag PT 2000 Divorce and extrapair mating in female Black-capped chickadees (*Parus atricapillus*): separate strategies with a common target. *Behav. Ecol. Sociobiol.* **49**, 18–23.

Saitou T. 2002 Factors affecting divorce in the Great Tit *Parus major*. *Ibis* **144**, 311–316.

Spiegelhalter DJ, Best NG, Carlin BR, van der Linde A. 2002 Bayesian measures of model complexity and fit. *J. Roy. Stat.l Soc. B-Stat. Method.* **64**, 583-616.

Valcu M, Kempenaers B. 2008 Causes and consequences of breeding dispersal and divorce in a Blue tit, *Cyanistes caeruleus*, population. *Anim. Behav.* **75**, 1949–1963.
